# Allergen-specific circulating CLA^+^ memory T cells stratify IL-22 response in atopic dermatitis skin

**DOI:** 10.3389/fimmu.2025.1599892

**Published:** 2025-07-01

**Authors:** Irene García-Jiménez, Lídia Sans-de San Nicolàs, Sandra Díez-Ribas, Laia Curto-Barredo, Marta Bertolín-Colilla, Ana Vivancos-Melenchón, Ignasi Figueras-Nart, Montserrat Bonfill-Ortí, Anna Ryzhkova, Marta Ferran, Tali Czarnowicki, Ramon M. Pujol, Luis F. Santamaria-Babí

**Affiliations:** ^1^ Immunologia Translacional, Departament de Biologia Cellular, Fisiologia i Immunologia, Facultat de Biologia, Universitat de Barcelona (UB), Parc científic de Barcelona (PCB), Barcelona, Spain; ^2^ Programa de Doctorat en Biomedicina, Universitat de Barcelona (UB), Barcelona, Spain; ^3^ Departament de Dermatologia, Hospital del Mar, Institut Hospital del Mar d’Investigacions Mèdiques (IMIM), Universitat Autònoma de Barcelona (UAB), Barcelona, Spain; ^4^ Departament de Dermatologia, Universitat de Barcelona (UB), L’Hospitalet de, Llobregat, Spain; ^5^ Dr. Phillip Frost Department of Dermatology and Cutaneous Surgery, University of Miami Miller School of Medicine, Miami, FL, United States

**Keywords:** atopic dermatitis, CLA^+^ memory T cells, epidermal thickness, house dust mite, IgE, IL-22, moderate-to-severe, stratification

## Abstract

**Background:**

Current understanding of IL-22 in atopic dermatitis (AD) mostly relies on animal models, intracellular staining of polyclonally activated peripheral lymphocytes, and biological therapies.

**Methods:**

We evaluated the IL-22 response to house dust mite (HDM) extract in 58 patients with moderate-to-severe AD using a coculture system made of circulating memory cutaneous lymphocyte associated antigen (CLA)^+/−^ T cells with autologous lesional epidermal cells. Additionally, we performed histological and gene expression analysis in lesional skin biopsies, assessed specific IgE levels in plasma, and together with the clinical features of the patients, were related to the IL-22 *in vitro* response.

**Results:**

HDM triggered heterogeneous IL-22 secretion in memory T cells, preferentially in the CLA^+^ subset, which enabled patient stratification into IL22 producers (IL22P, n=17) and non-producers (IL22NP, n=41). IL22P showed an increased degree of epidermal thickness, overexpression of IL22 in lesional skin areas, elevated specific IgE levels against HDM and SEB in plasma, and a higher proinflammatory profile compared to IL22NP.

**Conclusions:**

This is the first report showing that allergen-specific CLA^+^ T-cell-mediated IL-22 *in vitro* response functionally distinguish moderate-to-severe adult AD patients with specific clinical features and activated IL-22 pathway in their lesional skin, paving the way for the selection of patients that may benefit from IL-22-directed therapies.

## 1 Introduction

Interleukin-22 (IL-22), a cytokine of the IL-10 family, has emerged as a promising therapeutic target in atopic dermatitis (AD) (1, 2). This cytokine is produced by a wide range of immune cells including Th22, Th17, type 3 innate lymphoid cells, γδ T cells, natural killer cells and dendritic cells, although Th22 are known to be the main cellular source (3–5). These cells produce negligible or low levels of IL-17 and IFN-γ, and express the skin-homing receptors CCR4, CCR6, CCR10 and cutaneous lymphocyte-associated antigen (CLA) (6, 7).

The IL-22 receptor (IL-22R) consists of two subunits, IL-22Rα and IL-10Rβ, and primarily signals through the JAK/STAT pathway, among others. In the skin, its expression is limited to keratinocytes and dermal fibroblasts (5, 8). IL-22 adversely impacts epidermal structure by promoting keratinocyte proliferation while dampens terminal differentiation, resulting epidermal hyperplasia and barrier compromise (9, 10). Furthermore, this cytokine enhances the production of antimicrobial peptides such as β-defensins and S100A proteins (9, 10).

Data from animal models indicate that the overexpression of IL-22 in the skin conferred an AD-like phenotype characterized by chronic pruritus, thickening of the epidermis, skin barrier defects and increased susceptibility to *S. aureus* colonization (11, 12). In human studies, patients with AD show increased levels of serum IL-22, and elevated IL-22-producing T cells in peripheral blood and lesional skin compared to controls (3, 13–16). Moreover, IL-22 and IL-22R are highly upregulated in the skin of pediatric and adult patients with AD compared with normal skin and psoriatic lesions (3, 17). The administration of the anti-IL-22 monoclonal antibody fezakinumab to moderate-to-severe adult AD patients showed clinical and transcriptomic improvements in patients with high IL-22-baseline expression (2).

CLA^+^ T cells constitute a subset of memory T cells that reflect cutaneous abnormalities present in AD skin and represent a key player in the pathogenesis of the disease (18). Previous research of our group has shown that addressing the varied levels of relevant cytokines of the disease, including IL-13, IL-31 and IL-9, produced by CLA^+^ memory T cells in an ex vivo model of AD, is essential for stratifying patients based on clinical features like disease severity and pruritus (19–21).

Environmental factors, such as airborne allergens like house dust mite (HDM), are involved in AD exacerbation (22). Epicutaneous sensitization with HDM induces skin lesions with increased IL-22 mRNA expression and marked epidermal thickening in mice, dogs and humans (11, 23–25). HDM also induces the expression of IL-22Rα in keratinocytes and the production of IL-22 by T cells (26, 27). Interestingly, HDM-specific T cells infiltrating human AD lesions share TCR sequences with circulating CLA^+^ memory T cells (28).

While IL-22 is implicated in AD pathogenesis, the role of allergens in triggering IL-22 production by circulating CLA^+^ memory T cells remain unclear. To address this, we assessed the *in vitro* IL-22 response of allergen-specific CLA^+^ memory T cells from adults with moderate-to-severe AD, aiming to better define the role of IL-22 in AD and identify patient populations who may potentially benefit from IL-22–targeted therapies.

## 2 Materials and methods

### 2.1 Patients and biological samples

This study included 58 adults with moderate-to-severe AD and 17 non-age and non-sex-matched control individuals. All participants provided written informed consent, and sample collection was conducted according to the institutional review board-approved protocols at the Hospital del Mar and Hospital de Granollers (Spain). Clinical characteristics of patients and controls are summarized in **Supplementary Table S1**. Two skin biopsies, from lesional areas in AD patients, and blood tests were obtained without any topical or systemic anti-inflammatory treatments administered for a minimum of 2–4 weeks prior to the study, respectively. Lesional skin biopsies were used for different experimental procedures: a small portion for histological studies (n=28), another portion for RNA extraction (n=41) and the rest of the biopsy was used for the isolation of epidermal cell suspension (n=58). The workflow of the study design can be found in **Supplementary Figure S1**.

### 2.2 Isolation of circulating memory T cells and epidermal cell suspension

Circulating CLA^+^ and CLA^−^ memory CD45RA^−^ T lymphocytes were purified from whole blood after peripheral blood mononuclear cells (PBMC) isolation by Ficoll (GE Healthcare, Princeton, NJ, USA) gradient, and three successive immunomagnetic separations (Miltenyi Biotech, Bergisch Gladbach, Germany), as previously described (29). Skin biopsies were incubated overnight in Dispase (Corning, Corning, NY, USA) at 4°C. The epidermal sheet was peeled off from the dermis, cut into pieces, and incubated in trypsin (Biological Industries, Kibbutz Beit Haemek, Israel) for 15 min at 37°C. Epidermal tissue was mechanically disaggregated, and the cell suspension (Epi) was transferred to fresh media, as previously described (29).

### 2.3 Circulating memory T cells and epidermal cells coculture and activation

Cocultures of 5 × 10^4^ CLA^+/−^ T cells and 3 × 10^4^ autologous epidermal cells (CLA^+^/Epi or CLA^−^/Epi, respectively) in 96-well U-bottom plates (Falcon, Corning, NY, USA), were left untreated (M) or activated for 5 days with HDM extract (10 μg/mL) (kindly provided by LETI Pharma, Barcelona, Spain). Supernatants were collected and kept at −20°C for later cytokine quantification.

### 2.4 Cytokine quantification

ProcartaPlex multiplex immunoassays (Invitrogen, Waltham, MA, USA) were used to measure the concentration of IL-4, IL-5, IL-13, IL-17A, IL-22, IL-31 and IFN-γ in collected coculture supernatants with the MAGPIX plate reader (Luminex Technologies Inc., Austin, TX, USA). Data was analyzed with ProcartaPlex Analyst software version 1.0 (Invitrogen) using a five-parameter logistic curve. Values below the lower limit of quantification (LLOQ) were treated as zero.

### 2.5 Quantification of total and specific IgE against HDM and SEB

Total IgE (kU/L), HDM-specific IgE (response (OD)) and SEB-specific IgE (kU/L) plasma levels were measured by ImmunoCAP (ThermoFisher Scientific).

### 2.6 Histological analysis

Skin samples were fixed in 10% Formalin solution (Sigma-Aldrich, St. Louis, MO, USA) for 48h, embedded in paraffin, sectioned into 5-µm thick sections and stained with hematoxylin and eosin (H&E). Caseviewer software was used to evaluate epidermal thickness. In a spanning distance of 1,000 µm, four equidistant measurements were taken from the free margin of the skin to the epidermal ridge and dermal papillae. These values were averaged to give epidermal thickness value. All slides were analyzed by an examiner who was blinded to the identity of each sample.

### 2.7 RNA extraction and RT-qPCR

Total RNA, isolated from lesional skin tissue, was extracted with TRIzol (Invitrogen). cDNA was synthesized with the High-Capacity cDNA Reverse Transcription kit (Applied Biosystems, Waltham, MA, USA) and preamplified with the TaqMan PreAmp Master Mix (2x) (Applied Biosystems). Taqman Gene Expression Master Mix and FAM-labelled probes (**Supplementary Table S2**) were used for qRT-PCR in a QuantStudio 7 instrument, and data was processed by SDS analysis software version 2.4.1 (all Applied Biosystems). Expression levels were normalized to the human ribosomal protein *RPLP0*, as previously described (19).

### 2.8 Statistical analysis

Data analysis and representation were performed with GraphPad Prism software version 8 (GraphPad Software Corporation, San Diego, CA, USA). Data are generally represented as the median ± 95% confidence interval (CI). Data distribution was checked through Shapiro-Wilk test. Comparisons among groups IL22P, IL22NP and C were performed using the Kruskal-Wallis test, followed by pairwise comparisons using Wilcoxon and Mann-Whitney tests for paired and unpaired data, respectively. Multiple testing correction was applied to pairwise comparisons with the Benjamini & Hochberg false discovery rate (FDR) method. Correlations were examined using Spearman coefficient and represented with linear regression. Differences were considered significant at a P-value of less than .05 and represented by the following symbols: (*) p <.05; (**) p <.01; (***) p <.001; (****) p <.0001.

## 3 Results

### 3.1 HDM triggers heterogeneous IL-22 production preferentially in CLA^+^ memory T cells defining producer and non-producer patients with different epidermal hyperplasia in lesional skin

**Figure 1:**
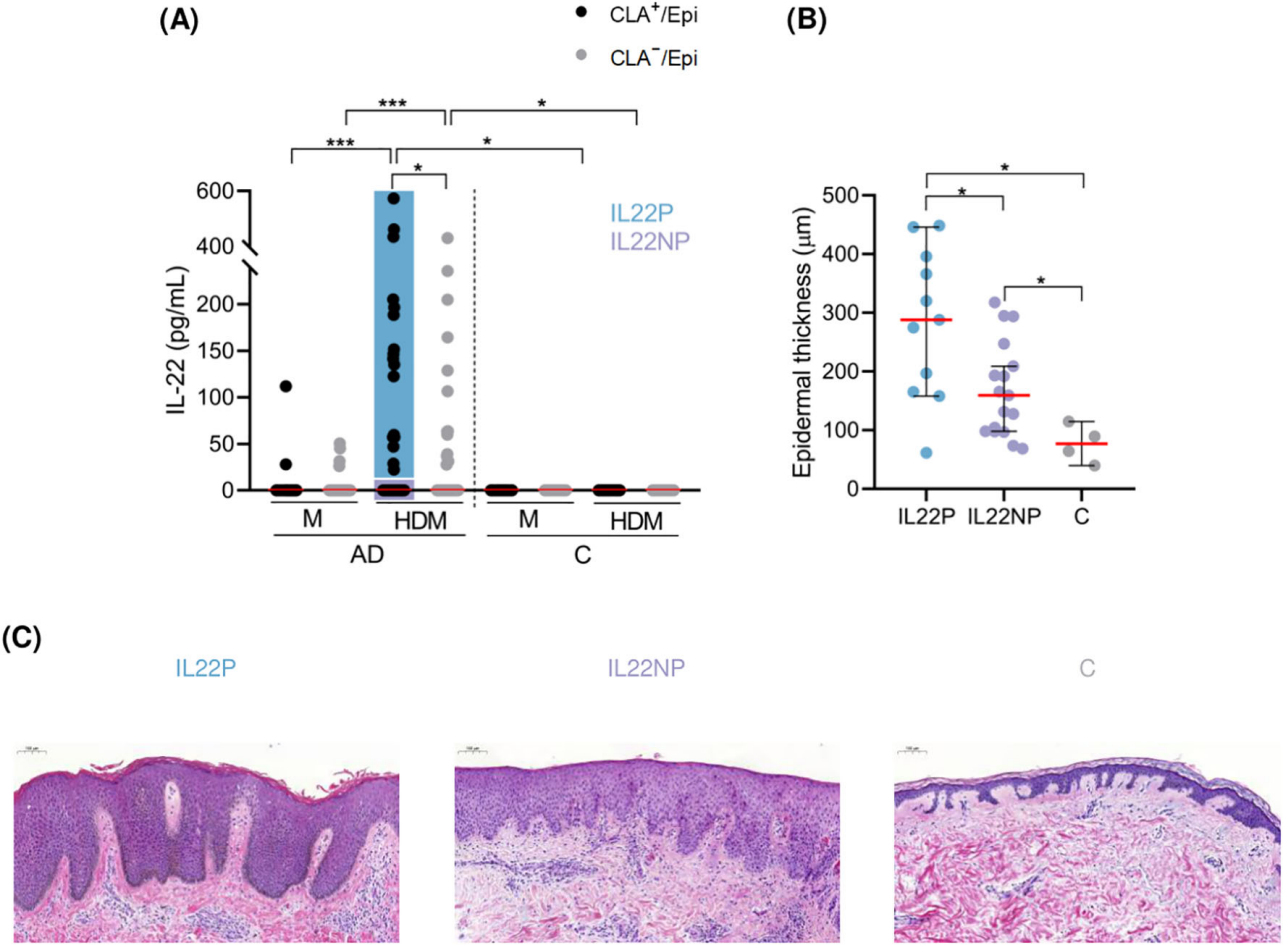
HDM-induced IL-22 by CLA^+^ memory T cells identify AD patients with increased degree of epidermal hyperplasia in their lesional skin. **(A)** AD patients were stratified according to IL-22 production by HDM-stimulated CLA^+^ memory T cells (IL22P (n=17), IL22NP (n=41)). **(B)** Epidermal thickness (µm) was compared between IL22P (n=11), IL22NP (n=17) and controls (n=4). **(C)** Representative H&E staining of skin sections from each group at magnification 10x. AD, atopic dermatitis; C, control subjects; CLA, cutaneous lymphocyte-associated antigen T cells; Epi, epidermal cell suspension; HDM, house dust mite; M, untreated. *p <.05; ***p <.001.

Circulating memory T-cell IL-22 production was assessed in AD patients (n=58) and control subjects (n=17). HDM activation resulted in significant induction of IL-22 in AD-derived cocultures, especially in those containing CLA^+^ over CLA^−^ memory T cells, and no IL-22 production was detected in control (C)-derived cocultures (**Figure 1A**). Furthermore, HDM-induced CLA^+^ memory T-cell-mediated IL-22 response was enhanced by the presence of autologous lesional epidermal cells (**Supplementary Figure S2A**), depended on HLA class II presentation, as observed using neutralizing antibodies (**Supplementary Figure S2B**) and relied on direct contact with lesional cell suspension (**Supplementary Figure S2C**), implying their feasibility as a source of antigen presenting cells.

IL-22 was detected in only 29% of the CLA^+^ T-cell cocultures, indicating a differentiated T-cell *in vitro* response to HDM extract among AD patients. To assess the possible effect of allergen-specific CLA^+^ T-cell IL-22 response on the morphology and molecular signature of lesional skin as well as the clinical profile of AD, patients were stratified based on IL-22 secretion into IL-22 producers (IL22P; n=17) and non-producers (IL22NP; n=41) (**Figure 1A**).

Epidermal thickness was measured in skin biopsies from AD lesions, and from corresponding sites in control subjects. We found that IL22P exhibited a significantly higher degree of epidermal thickening than IL22NP and controls (IL22P median = 288.00, IL22NP median = 159.50, C median = 76.90, IL22P *vs* IL22NP p = .022, IL22P *vs* C p = .022; **Figures 1B, C**). Although cytokines such as IL-4, IL-13, and IL-17A have been shown to induce epidermal hyperplasia (11, 30, 31), stratification of AD patients based on IL-4, IL-13, or IL-17A production by HDM-activated CLA^+^ memory T cells (**Supplementary Figure S3A**) was insufficient to account differences in epidermal thickness between subgroups (**Supplementary Figure S3B**).

### 3.2 Patients with CLA^+^ T-cell IL-22 response to HDM present elevated IL-22 expression in lesional skin compared to patients with no IL-22 secretion

**Figure 2:**
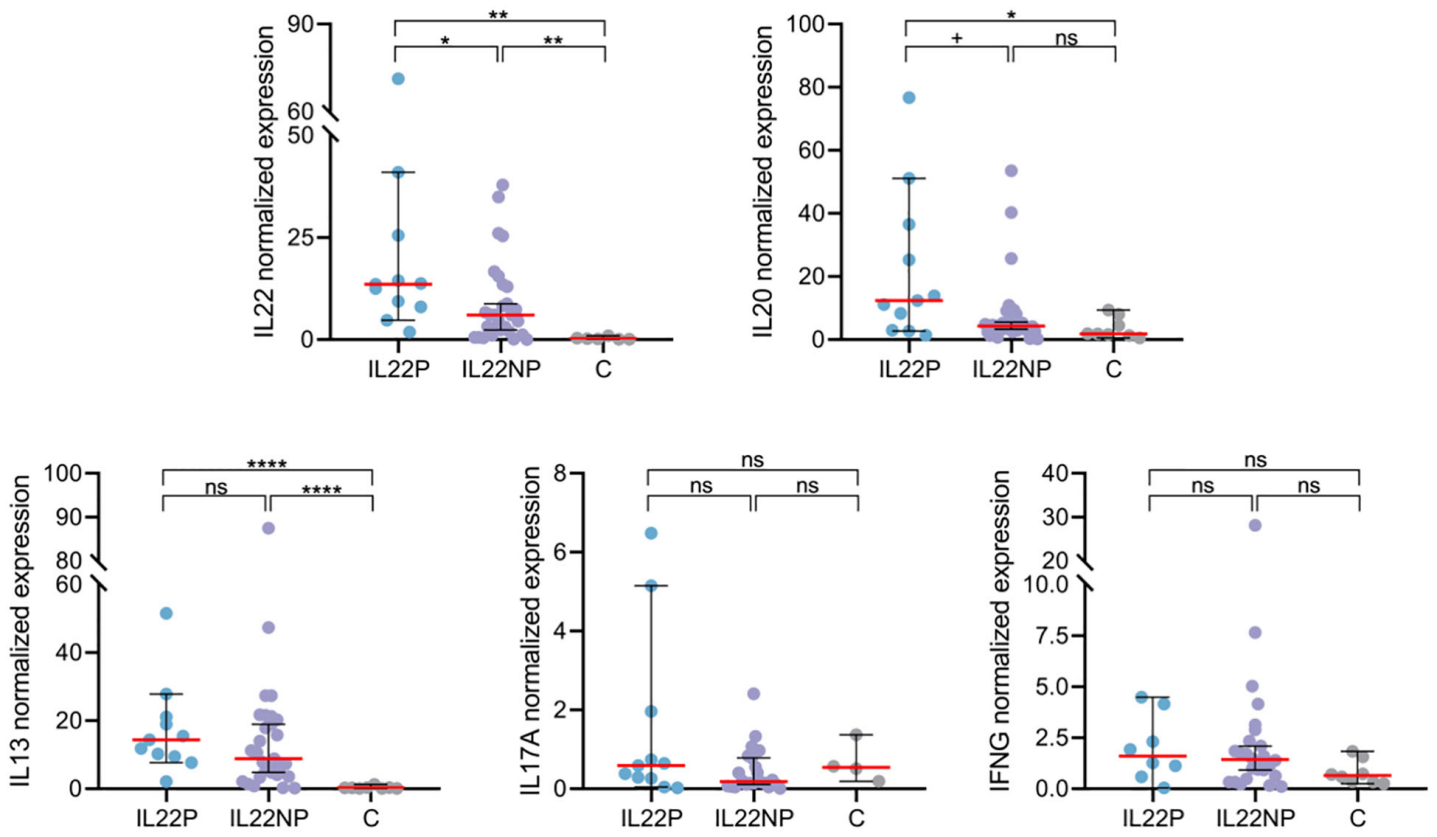
Lesional skin of IL22P patients exhibit marked IL-22 and IL-20 expression. Normalized expression of immune genes *IL22*, *IL20*, *IL13*, *IL17A* and *IFNG* in lesional skin biopsies was compared between IL22P (n=8-11), IL22NP (n=22-30) and controls (n=4-8). ns p >.1; ^+^p <.1; *p <.05; **p <.01; ****p <.0001.

To further investigate the underlying molecular mechanisms in IL22P skin, we quantified the expression of AD-associated cytokines including *IL22*, *IL20*, *IL13*, *IL17A* and *IFNG* using RT-qPCR in lesional skin from AD patients and skin from healthy controls. Among these cytokines, only *IL22* (IL22P median = 13.55, IL22NP median = 6.02, C median = 0.20, IL22P *vs* IL22NP p = .023, IL22P *vs* C p = .002), but not *IL13*, *IL17A* and *IFNG*, showed significant upregulation in IL22P compared with IL22NP and controls, and *IL20* followed the same trend (IL22P median = 12.40, IL22NP median = 4.30, C median = 1.81, IL22P *vs* IL22NP p = .051, IL22P *vs* C p = .027), (**Figure 2**).

### 3.3 IL-22 producers display an enhanced proinflammatory cytokine response and allergen sensitization

**Figure 3:**
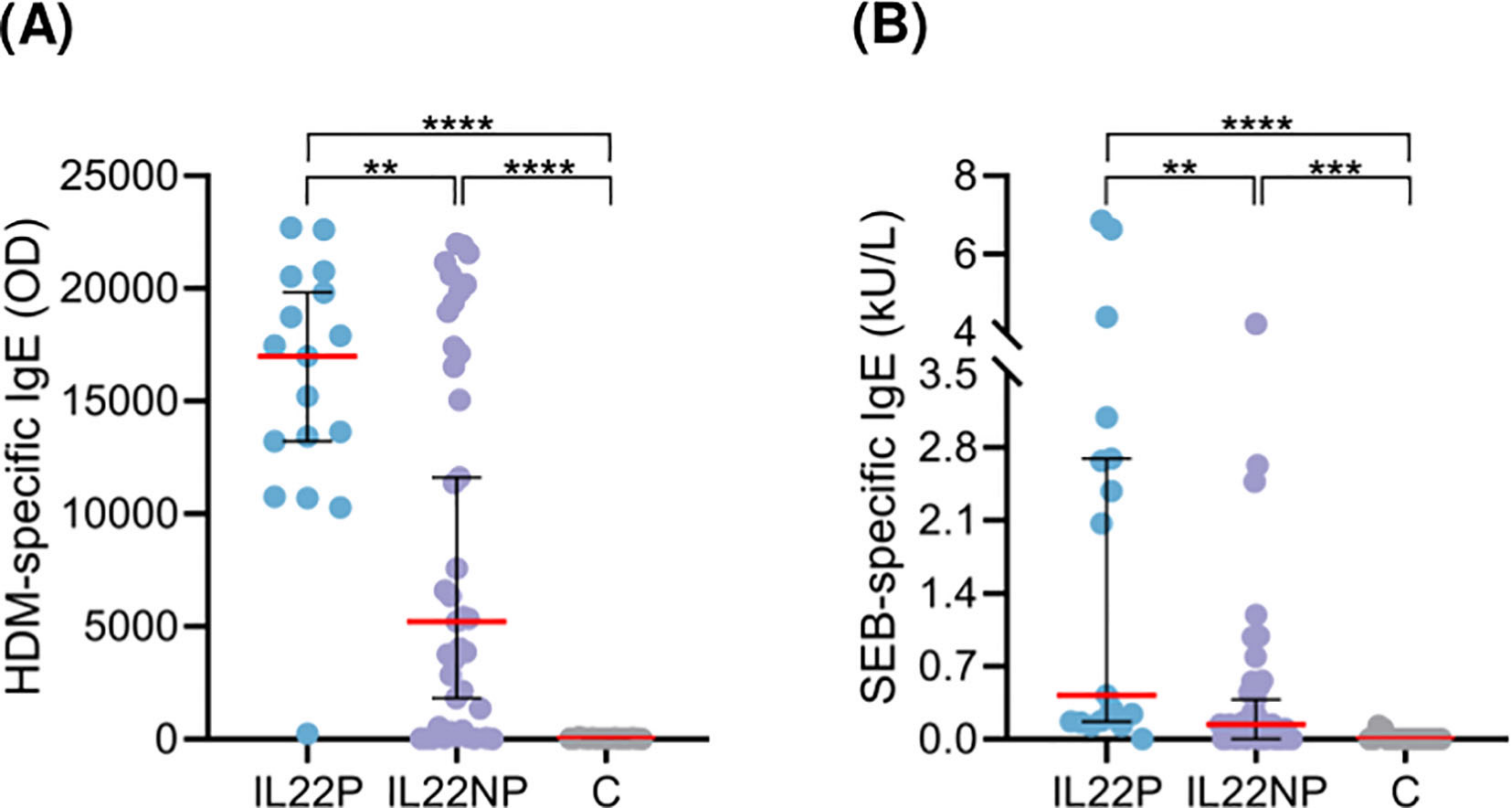
Patients producing IL-22 present elevated HDM- and SEB-specific IgE levels in plasma. Plasma levels of specific IgE against **(A)** HDM (OD) and **(B)** SEB (kU/L) were compared between IL22P (n=17), IL22NP (n=35-41) and controls (n=17). C, control subjects; HDM, house dust mite; OD, optical density; SEB, staphylococcal enterotoxin **(B)** **p <.01; ***p <.001; ****p <.0001.

IL22P exhibited significantly higher HDM- and SEB-specific IgE levels in plasma compared to IL22NP and controls (**Figures 3A, B**). However, no significant differences were observed in demographic, epidemiological, or clinical characteristics between the AD groups (**Supplementary Table S3**).

Interestingly, when AD patients were stratified according to their capacity to secrete IL-22 by HDM-activated CLA^−^ memory T cells, no difference in epidermal hyperplasia or specific IgE levels were observed between the two AD groups (**Supplementary Figures S4A-D**).

**Figure 4:**
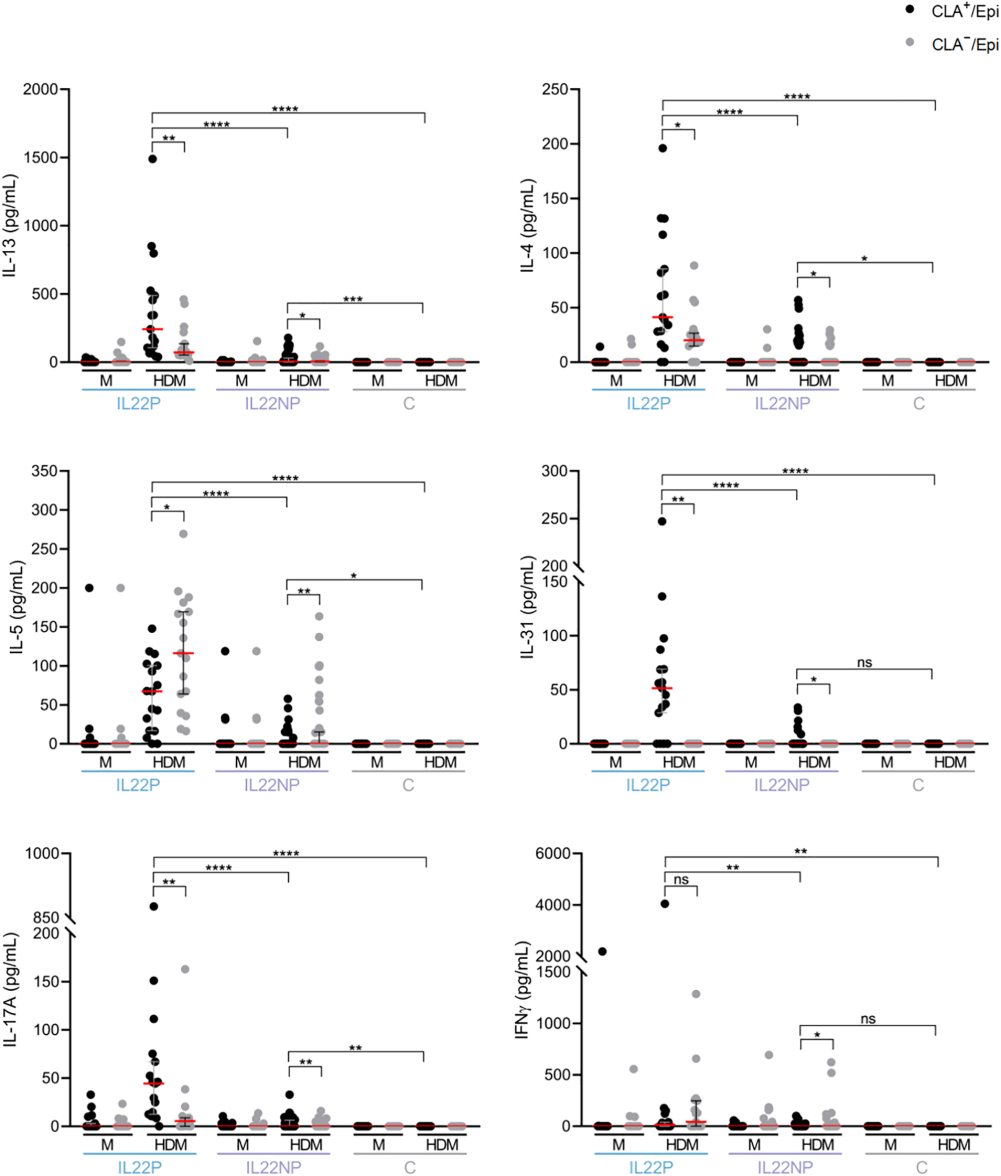
Under HDM stimulation, IL22P secrete significantly higher levels of Th2 cytokines, IL-17A and IFN-γ. IL-13, IL-4, IL-5, IL-31, IL-17A and IFN-γ cytokines were simultaneously quantified in CLA^+^/Epi and CLA^-^/Epi cocultures of IL22P (n=17) and IL22NP (n=41) AD patients and control subjects (n=17). AD, atopic dermatitis; C, control subjects; CLA, cutaneous lymphocyte-associated antigen T cells; Epi, epidermal cell suspension; HDM, house dust mite; M, untreated. ns p >.05; *p <.05; **p <.01; ***p <.001; ****p <.0001.

As shown in **Figure 4**, the cytokine profiles of HDM-stimulated CLA^+^ T-cell cocultures differed markedly between groups. IL-22–producing (IL22P) patients exhibited significantly higher levels of IL-13, IL-4, IL-5, IL-31, IL-17A, and IFN-γ compared to IL-22 non-producers (IL22NP) and healthy controls, indicating a heightened proinflammatory response in this subgroup. Notably, although IL22NP patients did not produce IL-22 in response to HDM, they still showed elevated production of Th2, Th17, and Th1 cytokines—specifically IL-13, IL-4, IL-5, and IL-17A—compared to controls.

In parallel, IL22NP patients demonstrated a milder disease phenotype than IL22P patients, with reduced epidermal thickening and lower sensitization to SEB and HDM. However, relative to healthy controls, IL22NP patients still presented with increased epidermal hyperplasia and higher allergen-specific IgE levels (**Figure 1B**, **3B, C**), consistent with their diagnosis of AD.

## 4 Discussion

While the role of IL-22 in driving epidermal hyperplasia and barrier dysfunction in AD is well established, the functional stratification of IL-22–producing patients with moderate-to-severe AD in response to a disease-relevant trigger has not been previously described. Our results show that the heterogenous IL-22 production induced by HDM-stimulated circulating CLA^+^ memory T cells allow the identification of patients with increased epidermal hyperplasia, elevated *IL22* mRNA expression in lesional skin, and a marked sensitization status to HDM and SEB.

Currently, most of the studies use intracellular flow cytometry of polyclonal-activated lymphocytes from peripheral blood and skin biopsies to investigate T-cell-derived IL-22 secretion in AD (3, 4, 16, 32). Although polyclonal activators are valuable tools to understand T-cell activation in mechanistic studies, clinical triggers serve as more suitable models for evaluating T-cell responses in the setting of disease. Several studies have investigated the effects of physiological drivers of AD, including aeroallergens, on IL-22 induction in T cells (26, 27). However, the relationship between IL-22 production and the clinical context or inter-individual variability among patients remains unexplored.

Given the capacity of our ex vivo model of AD—based on cocultures of circulating memory T cells and autologous lesional epidermal cells—to generate translational insights by linking CLA^+^ T-cell cytokine production with patient clinical features (19–21), we used this system to investigate the IL-22 response. We found that the secretion of IL-22 by CLA^+^ memory T cells in response to HDM was heterogeneous and helped to define two groups: AD patients producing IL-22 (IL22P) and those with no IL-22 production (IL22NP). Consistent with the known role of IL-22 in promoting epidermal hyperplasia, skin biopsies from AD lesional from IL22P showed an increased degree of epidermal thickening in comparison to the IL22NP group and to controls. While HDM stimulation primarily induced IL-22 responses in CLA^+^ skin-homing memory T cells, it also triggered heterogeneous IL-22 production in CLA^-^ memory T cells, albeit to a lesser extent. This response may still contribute to the initiation of the atopic march by inducing systemic T-cell activation with the potential to migrate beyond the skin and promote inflammation in other organs.

The co-occurrence of other cytokines in the IL22P group may support the multicytokine nature of AD, the existence of double-positive cytokine T cells, and potential crosstalk between cytokine axes that may augment each other’s effects. This interaction likely contributes to skin barrier dysfunction, thereby facilitating the persistence of AD and promoting the progression of the atopic march.

Although CLA^+^ memory T cells from IL22NP do not produce IL-22 upon HDM activation, they respond to HDM and secrete significantly more IL-13, IL-4, IL-5 and IL-17 compared to controls. Clinically, IL22NP patients share similar features with IL22P patients, except for lower levels of HDM- and SEB-specific IgE. These findings suggest that IL22NP patients represent a distinct immunological profile compared to both IL22P and healthy individuals, potentially reflecting a separate AD endotype, the clinical significance of which remains to be defined.

Because of an abnormal skin barrier, along with immune dysregulation, AD skin is susceptible to *S. aureus* colonization and allergen sensitization (33–35). In fact, AD patients colonized by *S. aureus* exhibited major barrier dysfunction and allergen sensitization than non-colonized patients (36). Although we did not assess the presence of *S. aureus* in the skin of our participants—a potential limitation of the study—previous reports have demonstrated high levels of *S. aureus* and SEB exposure in AD patients (37–40). Consistent with the known role of IL-22 in impairing the skin barrier, we observed notably elevated SEB-specific IgE levels in IL22P patients. Additionally, this AD group also exhibited elevated plasma IgE levels against HDM, reinforcing the notion that a compromised skin barrier increases susceptibility to pathogen penetration.

A previous murine study demonstrated that skin-specific expression of IL-22 increased barrier permeability, promoted *S. aureus* colonization, and exacerbated AD-like inflammation upon allergen exposure (11). Our findings may represent a human correlate, as we show activation of the IL-22 pathway in the lesional skin of IL22P patients. Our observations in the IL22P AD subgroup align with the immunopathological effects of IL-22 described in the transgenic mouse model and support the functional stratification into IL22P and IL22NP.

Our study has some limitations. We did not study other frequent sensitizing allergens in AD which may also contribute to the total IL-22 found in patients. Sample size for both histological and RT-qPCR analysis was limited. We detected a significant increase in the degree of epidermal hyperplasia and *IL22* expression in skin lesions and a marked sensitization to HDM and SEB in IL22P patients. However, these clinical, histological and molecular features associated with the IL22P phenotype deserve to be further validated in a larger independent cohort of AD patients.

IL-22 is clinically relevant in AD, as demonstrated by a phase II clinical trial in which treatment with an anti–IL-22 monoclonal antibody led to significant improvement in adults with moderate-to-severe chronic AD, particularly in those with high baseline IL-22 expression (2). New directed therapies that inhibit IL-22 signaling are emerging, such as temtokibart, a mAb that targets the IL22RA1, which is showing promising efficacy with minimal risk (41, 42). Additionally, dupilumab—though not directly targeting IL-22—has been shown to decrease IL-22 mRNA expression in lesional skin and reduce IL-22 protein levels in serum and tissue. These effects are associated with clinical improvement and reduced epidermal hyperplasia (43, 44). Therapies targeting other immune pathways—such as the OX40-OX40L axis—are also being explored. Amlitelimab, an OX40-OX40L pathway inhibitor, has been shown to reduce serum levels of IL-22, IL-13, and IL-31, offering a potential treatment option for AD patients with dominant Th2/Th22-driven inflammation (45).

Taken together, our findings suggest that studying IL-22 production by allergen-specific CLA^+^ memory T cells can be a useful approach to identify a subgroup of AD patients with an activated IL-22 pathway in lesional skin, who could benefit from IL-22–targeted therapies. This may be particularly relevant in immunologically blended AD endotypes, such as those observed in children. Further characterization of these patient subgroups using multi-omics approaches will be essential for discovering robust biomarkers to aid in the identification of individuals most likely to respond to IL-22–directed treatment strategies.

## Data Availability

The original contributions presented in the study are included in the article/**Supplementary Material**, further inquiries can be directed to the corresponding author.
