# Supplementary material for: Allergen-specific circulating CLA+ memory T cells stratify IL-22 response in atopic dermatitis skin
Source: Front Immunol. 2025 Jul 1;16:1599892. doi: 10.3389/fimmu.2025.1599892 (PMC12259416; doi:10.3389/fimmu.2025.1599892)
Supplement: Supplementary file 3 [file Presentation1.pptx]

## Slide 1
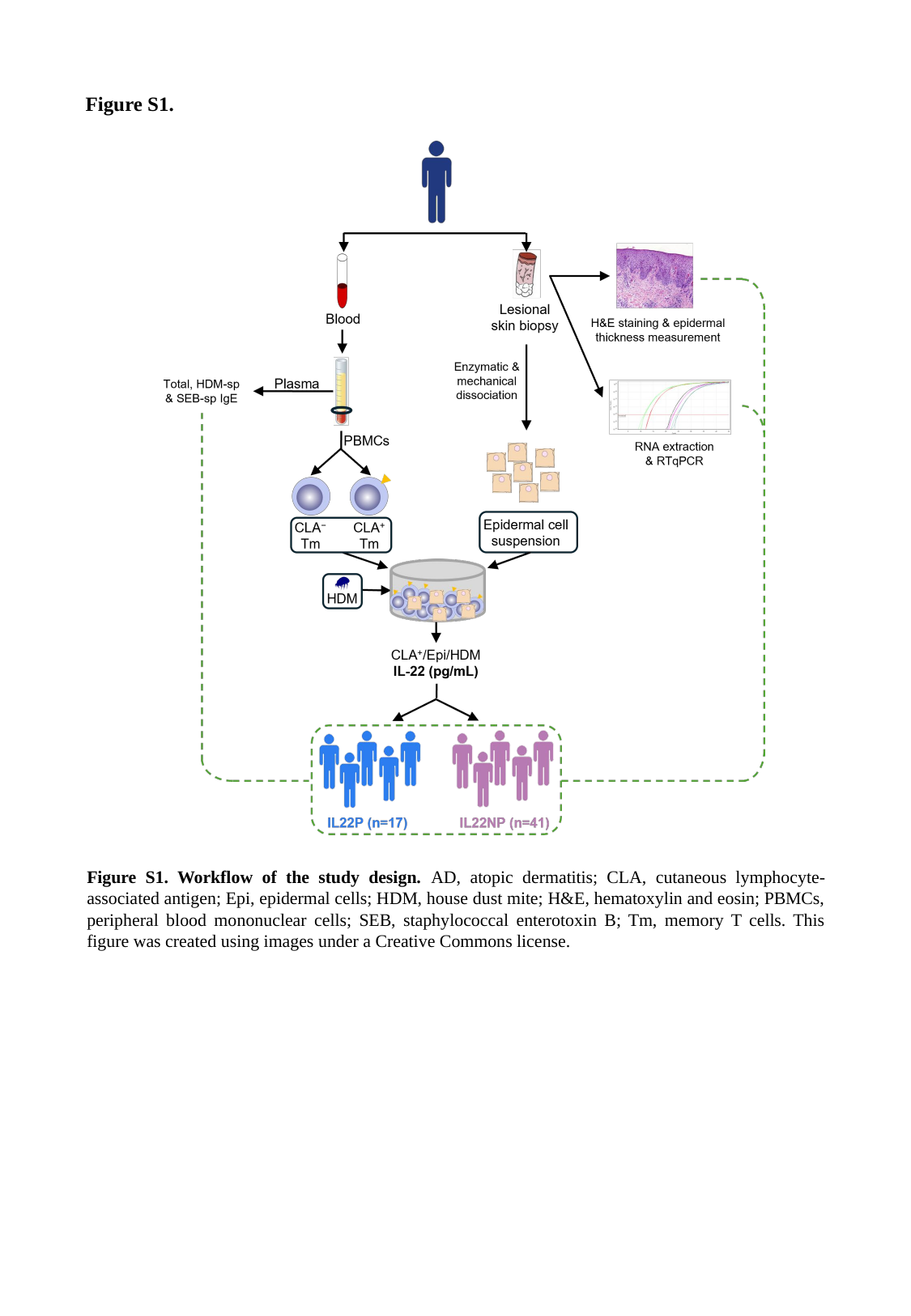

Figure S1.
Figure S1. Workflow of the study design. AD, atopic dermatitis; CLA, cutaneous lymphocyte-associated antigen; Epi, epidermal cells; HDM, house dust mite; H&E, hematoxylin and eosin; PBMCs, peripheral blood mononuclear cells; SEB, staphylococcal enterotoxin B; Tm, memory T cells. This figure was created using images under a Creative Commons license.

## Slide 2
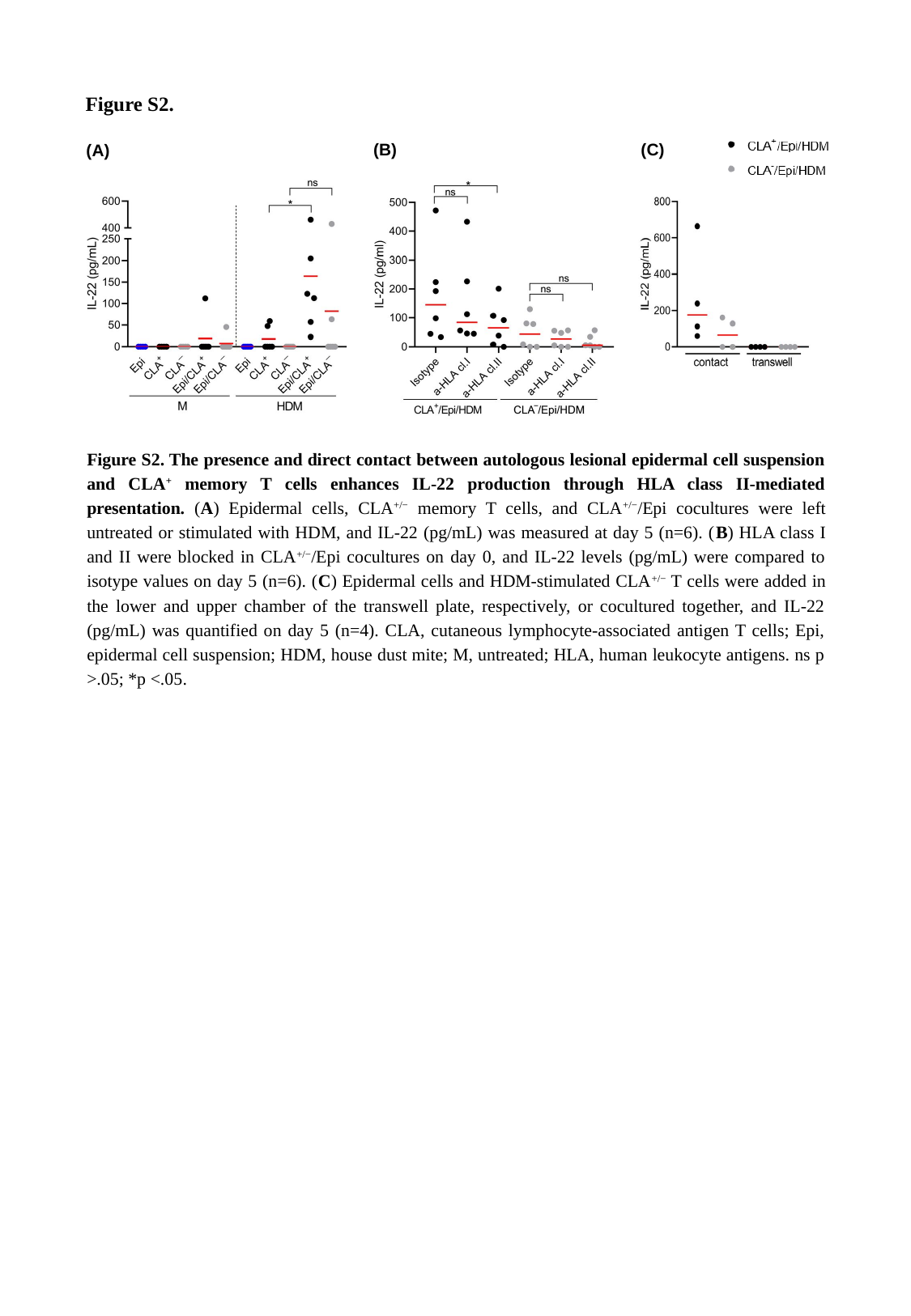

Figure S2.
(B)
(C)
(A)
Figure S2. The presence and direct contact between autologous lesional epidermal cell suspension and CLA+ memory T cells enhances IL-22 production through HLA class II-mediated presentation. (A) Epidermal cells, CLA+/− memory T cells, and CLA+/−/Epi cocultures were left untreated or stimulated with HDM, and IL-22 (pg/mL) was measured at day 5 (n=6). (B) HLA class I and II were blocked in CLA+/−/Epi cocultures on day 0, and IL-22 levels (pg/mL) were compared to isotype values on day 5 (n=6). (C) Epidermal cells and HDM-stimulated CLA+/− T cells were added in the lower and upper chamber of the transwell plate, respectively, or cocultured together, and IL-22 (pg/mL) was quantified on day 5 (n=4). CLA, cutaneous lymphocyte-associated antigen T cells; Epi, epidermal cell suspension; HDM, house dust mite; M, untreated; HLA, human leukocyte antigens. ns p >.05; *p <.05.

## Slide 3
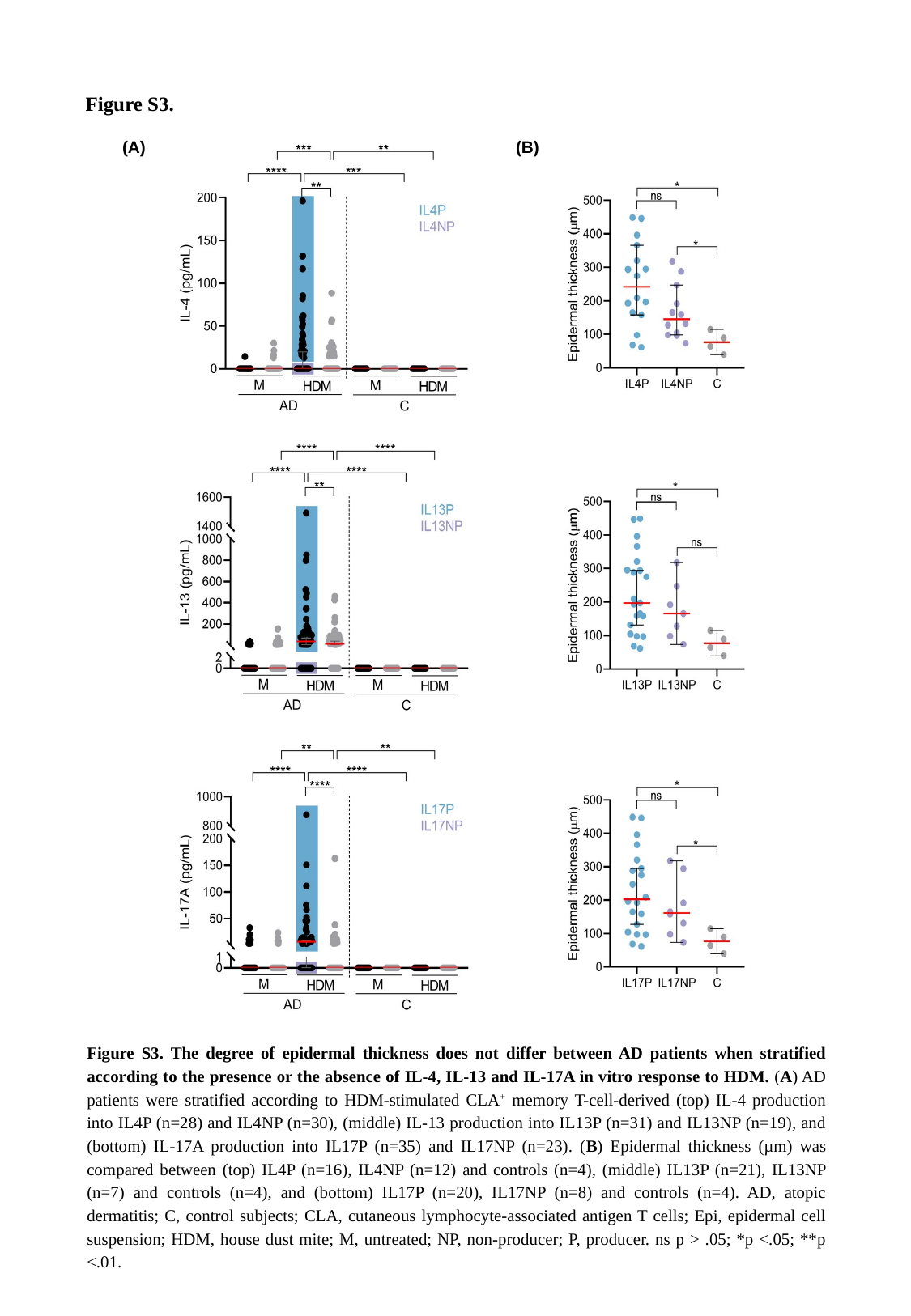

Figure S3.
(A)
(B)
Figure S3. The degree of epidermal thickness does not differ between AD patients when stratified according to the presence or the absence of IL-4, IL-13 and IL-17A in vitro response to HDM. (A) AD patients were stratified according to HDM-stimulated CLA+ memory T-cell-derived (top) IL-4 production into IL4P (n=28) and IL4NP (n=30), (middle) IL-13 production into IL13P (n=31) and IL13NP (n=19), and (bottom) IL-17A production into IL17P (n=35) and IL17NP (n=23). (B) Epidermal thickness (µm) was compared between (top) IL4P (n=16), IL4NP (n=12) and controls (n=4), (middle) IL13P (n=21), IL13NP (n=7) and controls (n=4), and (bottom) IL17P (n=20), IL17NP (n=8) and controls (n=4). AD, atopic dermatitis; C, control subjects; CLA, cutaneous lymphocyte-associated antigen T cells; Epi, epidermal cell suspension; HDM, house dust mite; M, untreated; NP, non-producer; P, producer. ns p > .05; *p <.05; **p <.01.

## Slide 4
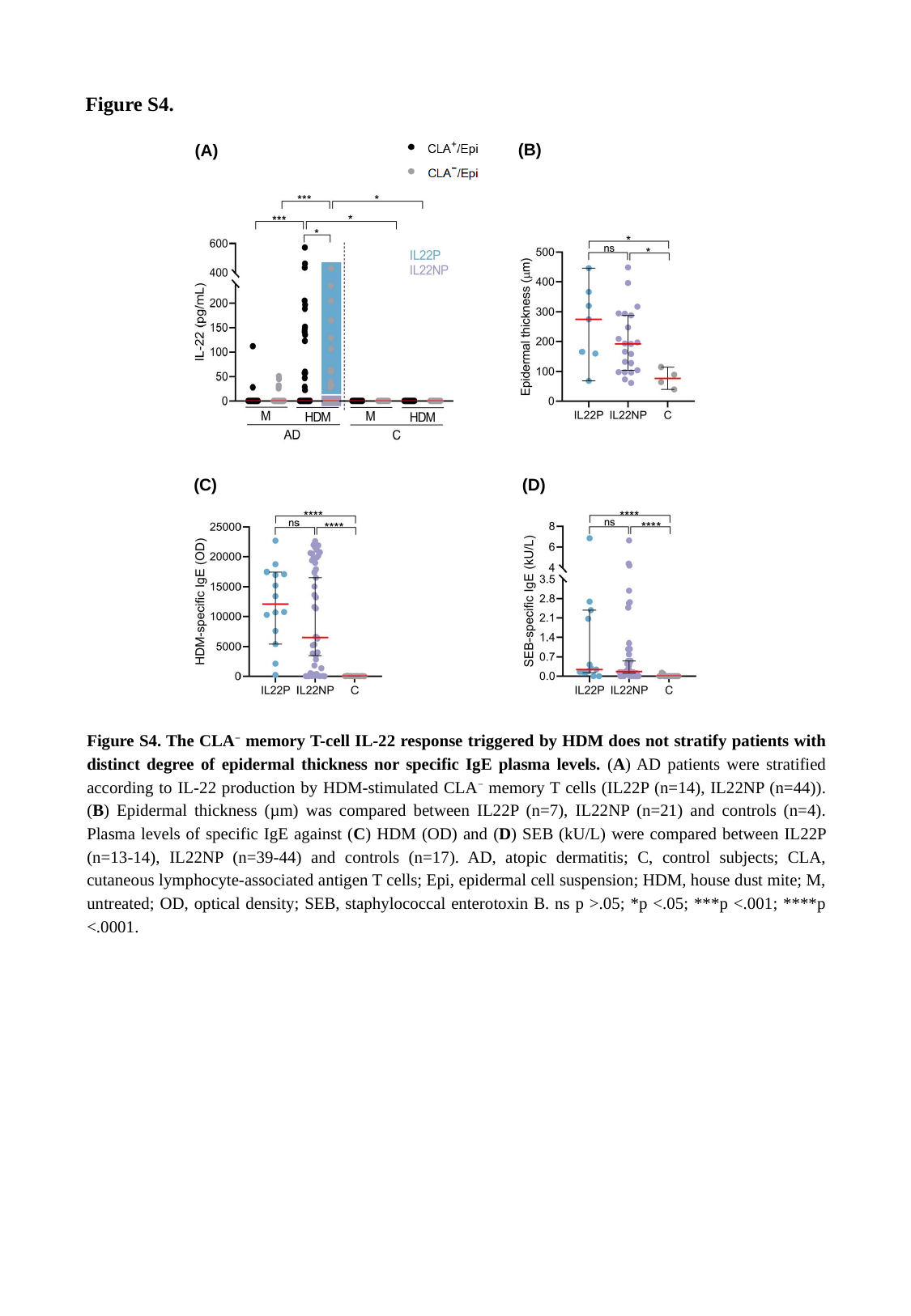

Figure S4.
(B)
(A)
(C)
(D)
Figure S4. The CLA− memory T-cell IL-22 response triggered by HDM does not stratify patients with distinct degree of epidermal thickness nor specific IgE plasma levels. (A) AD patients were stratified according to IL-22 production by HDM-stimulated CLA− memory T cells (IL22P (n=14), IL22NP (n=44)). (B) Epidermal thickness (µm) was compared between IL22P (n=7), IL22NP (n=21) and controls (n=4). Plasma levels of specific IgE against (C) HDM (OD) and (D) SEB (kU/L) were compared between IL22P (n=13-14), IL22NP (n=39-44) and controls (n=17). AD, atopic dermatitis; C, control subjects; CLA, cutaneous lymphocyte-associated antigen T cells; Epi, epidermal cell suspension; HDM, house dust mite; M, untreated; OD, optical density; SEB, staphylococcal enterotoxin B. ns p >.05; *p <.05; ***p <.001; ****p <.0001.
